# Identification and *In Vitro* Derivation of Spermatogonia in Beagle Testis

**DOI:** 10.1371/journal.pone.0109963

**Published:** 2014-10-15

**Authors:** Kyung Hoon Lee, Ran Lee, Won Young Lee, Dong Hoon Kim, Hak Jae Chung, Jin Hoi Kim, Nam Hyung Kim, Suk Hwa Choi, Jae Hwan Kim, Hyuk Song

**Affiliations:** 1 Department of Animal and Food Bioscience, RIBH, Konkuk University, Chung-ju, Korea; 2 Animal Biotechnology Division, National Institute of Animal Science, Suwon, Korea; 3 Department of Animal Biotechnology, Konkuk University, Seoul, Korea; 4 Department of Animal Science, Chungbuk National University, Choung-ju, Korea; 5 Department of Veterinary Science, Chungbuk National University, Choung-ju, Korea; 6 Departmemt of Biomedical Science, CHA University, Seongnam, Republic of Korea; Institute of Zoology, Chinese Academy of Sciences, China

## Abstract

**Background:**

In vitro culture of spermatogonial stem cells (SSCs) is important for exploration of SSCs self-renewal, differentiation, and manipulation. There are several reports on rodent SSC cultures; however, data on SSC cultures in domestic animals are limited. To provide basic scientific information on canine SSC cultures, we report canine testes development, and the development of spermatogonia-derived colonies (SDCs) for in vitro cultures.

**Methodology/Principal Findings:**

Testes from 2-, 3-, and 12-month-old beagles were used for histology, immunohistochemistry, in vitro culture, immunocytochemistry, and PCR. Protein gene product 9.5 (PGP9.5)-positive spermatogonia, both single and paired, were found to be abundant in the testes of 2-month-old beagles. stempro-34 and Dulbecco's modified Eagle medium with 5% fetal bovine serum provided as useful substrates for culture of SDCs, and fibroblast growth factor (FGF) played a key role in colony formation. Colonies were positive for alkaline phosphatase and anti-PGP9.5 staining. The early spermatogonia and stem cell markers such as octamer binding protein 4 (*Oct4*), Nanog homeobox (*Nanog*), promyelocytic leukemia zinc finger (*PLZF*), *PGP9.5*, and GDNF family receptor alpha-1 (*GFRα-1*) were expressed in the colonies at higher levels than in the testis tissue.

**Conclusions:**

Testes of the 2-month-old beagles had abundant single and paired spermatogonia, which can be used for derivation of SDCs, and FGF was important for colony formation.

## Introduction

Spermatogenesis, or mitosis and meiosis of male germ cells, is a dynamic event that occurs only in testicular seminiferous tubules when spermatogonia transform into spermatozoa through the complex biological process of cellular transformation. In mice, the seminiferous epithelium cycle is divided into 12 stages [1]. Unlike mice, the canine seminiferous epithelium cycle is divided into 8 stages that are determined by development of the acrosome system; the morphology of developing spermatids; as well as tubular morphology, in which the shape and location of the spermatid nuclei are considered the main aspects [2], [3]. Spermatogonia are important for the maintenance of spermatogenesis, and several spermatogonia markers have been reported in rodents [4], [5]. In the canine embryonic stage, OCT4-positive spermatogonia were detected in primitive gonads along with a paramesonephric region and primitive kidney [6]. In canine adult testes, NANOG was specifically expressed in spermatocytes and round spermatids at stages 6 through 8 and stages 1 through 5, respectively [7]. KIT expression has been observed in interstitial Leydig cells and spermatogonia of adult canine testes [8]. However, stage-specific spermatogonia markers remain to be identified in neonatal canine testes [9]. In addition, distribution of spermatogonia in the seminiferous tubules during growth-dependent testes development is not known.

Transplantation of rodents, and some domestic animal spermatogonial stem cells (SSCs), can colonize in the testes of recipient mice, making them fertile through spermatogenesis of the donor-derived spermatogonia. SSCs can be maintained by long-term cultures [10], [11], [12]. In rodents, it has been reported that spermatogenesis can be induced by a soft agar culture system by using SSCs from testis tissue fragments [13], [14]. In canines, donor-derived sperms have been produced in testes of the recipient dog after SSC transplantation [15]. However, a canine spermatogonia in vitro culture system has not been established, unlike those reported for mice and pigs [12], [16], [17]. The purpose of this study was to understand the development of canine testes, and to develop a spermatogonia-derived colony (SDC) in vitro culture system.

## Materials and Methods

### Canine testes preparation

Nine beagles (2-, 3-, and 12-months old; n = 3 each) were purchased from ORIENT (Seongnam, South Korea). Body weight was measured and testes were obtained after castration at Cheongbuk National University. The testes were fixed in Bouin's solution (Sigma-Aldrich, St. Louis, MO, USA) for histology and immunohistochemistry. The testes were decapsulated for spermatogonia culture. The study protocol and standard operating procedures were reviewed and approved by the Institutional Animal Care and Use Committee of Chungbuk National University and Konkuk University (Permit Number: CBNUA-601-13-01).

### Histology and immunohistochemistry

Neonatal testes were cut into three parts, fixed in Bouin's solution, dehydrated in ethanol, and embedded in paraffin. Six-micrometer-thick sections were cut with a microtome and stained with hematoxylin-eosin. After mounting sections under a cover glass, slides were observed using a light microscope (Carl Zeiss, Oberkochen, Germany); the magnification used was ×400 and ×1000. Conventional immunohistochemistry was performed. Briefly, the 6-µm-thick sections were deparaffinized with xylene and then dehydrated with ethanol. The slides were incubated with a target unmasking fluid (Accurate Chemical & Scientific Corp., Westbury, NY, USA) for 15 min, using a microwave oven to retrieve the antigens. The slides were washed three times with phosphate-buffered saline (PBS) and treated with 3% peroxide to block endogenous peroxidase; next, 1% (v/v) bovine serum albumin (BSA) was used as a blocking solution. The slides were incubated with anti-protein gene product 9.5 (PGP9.5, 1∶500; AbD Serotec, Raleigh, NC, USA), GDNF family receptor alpha-1 (GFRα-1, 1∶50; Santa Cruz Biotechnology, Dallas, TX, USA), promyelocytic leukemia zinc finger (PLZF, 1∶50; Santa Cruz Biotechnology, Dallas, TX, USA), Nanog homeobox (Nanog, 1∶100; Millipore, Billerica, MA, USA), deleted in azoospermia-like (DAZL, 1∶50; Santa Cruz Biotechnology, Dallas, TX, USA), OCT4 (1∶50, Santa Cruz Biotechnology, Dallas, TX, USA), spermatogenesis and oogenesis specific basic helix-loop-helix 1 (Sohlh1, 1∶50, Santa Cruz Biotechnology, Dallas, TX, USA), thymus cell antigen 1 (Thy1, 1∶50; Santa Cruz Biotechnology, Dallas, TX, USA), and CD9 (1∶50, Santa Cruz Biotechnology, Dallas, TX, USA) for 1 h at room temperature (RT), and then washed three times with PBS. Some of the slides were incubated with 1% BSA as a negative control. Slides were incubated with horse radish peroxidase-conjugated secondary antibody (1∶500; Santa Cruz Biotechnology, Dallas, TX, USA) for 1 h at RT (25°C), followed by incubation in 3,3′-diaminobenzidine (Vector Laboratories, Burlingame, CA, USA). The slides were washed with PBS and then observed under a light microscope (Carl Zeiss, Oberkochen, Germany) at a magnification of ×200 and ×400.

### Cell culture

Total testicular cells were prepared as previously described [17]. Testes from beagles were used for spermatogonial collection. Enzyme A (0.5 mg/ml collagenase, 0.01 mg/ml DNAse I, 0.1 mg/ml soybean trypsin inhibitor, and 0.1 mg/ml hyaluronidase) was added at a five-fold volume of enzyme to tissue (v/w) to decapsulated testes and incubated for 10 min at RT. The testes were washed with PBS, and then enzyme B (10 mg/ml collagenase, 0.01 mg/ml DNAse I, and 0.1 mg/ml soybean trypsin inhibitor) was added at a five-fold volume of original testes weight (v/w) and incubated for 15 min at RT. The testes were washed with 10% serum replacement for inactivation of the enzyme (Gibco, Carlsbad, CA, USA) in PBS. The enzyme-treated testes were meshed using a 40-µm nylon mesh, and red blood cells (RBCs) were eliminated using RBC lysis buffer (Sigma-Aldrich, St Louis, MO, USA). The isolated cells were seeded onto 0.2% (w/v) gelatin-coated 12-well plates (2×10^5^ cells/well) and incubated at 37°C in 5% CO_2_ for 7 days. Stempro-34 medium (Gibco, Carlsbad, CA, USA) and Dulbecco's modified Eagle medium (DMEM; Gibco, Carlsbad, CA, USA) with fetal bovine serum (FBS) were used for in vitro culture of canine spermatogonia. The stempro-34 medium was supplemented with insulin-transferrin-selenium (ITS; 25 µg/ml, 100 µg/ml, or 30 nM), 6 mg/ml glucose, 2 mM l-glutamine, 1% NEAA solution, 1% vitamin solution, 100 U/ml Penicillin/Streptomycin, 1 mM sodium pyruvate, 0.1 mM vitamin C, 1 µg/ml lactic acid, 30 ng/ml estradiol, 60 ng/ml progesterone, 0.2% BSA, and 1% knockout serum replacement. FBS was added at concentrations of 1%, 5%, or 10% to DMEM containing 2 mM l-glutamine, 0.1 mM β-mercaptoethanol, 1% NEAA solution, 100 U/ml of penicillin, and 100 µg/ml of streptomycin. Combinations of 20 ng/ml mouse epidermal growth factor (mEGF), 10 ng/ml basic fibroblast growth factor (bFGF), 10 ng/ml glial cell-derived neurotrophic factor (GDNF), and 10^3^ U/ml leukemia inhibitory factor (LIF) were applied to each medium for canine spermatogonia culture (Table 1). After 7 days, colonies were analyzed for PCR, immunocytochemistry and alkaline phosphatase staining.

**Table 1:** Media used with growth factors in total testicular cell culture.

Group no.	Maim medium	Added growth factors
1	Stempro-34	Without growth factors
2	Stempro-34	GDNF
3	Stempro-34	GDNF, FGF
4	Stempro-34	GDNF, FGF, LIF, EGF
5	DMEM+1% FBS	Without growth factors
6	DMEM+1% FBS	GDNF
7	DMEM+1% FBS	GDNF, FGF
8	DMEM+1% FBS	GDNF, FGF, LIF, EGF
9	DMEM+5% FBS	Without growth factors
10	DMEM+5% FBS	GDNF
11	DMEM+5% FBS	GDNF, FGF
12	DMEM+5% FBS	GDNF, FGF, LIF, EGF
13	DMEM+10% FBS	Without growth factors
14	DMEM+10% FBS	GDNF
15	DMEM+10% FBS	GDNF, FGF
16	DMEM+10% FBS	GDNF, FGF, LIF, EGF

### Immunocytochemistry

Colonies in 12-well plates were fixed with 4% paraformaldehyde (PFA, w/v) in PBS and washed with PBS. For permeabilization, the colonies were incubated with PBS containing 0.25% Triton X-100 (PBST) for 15 min and then washed with PBS. The colonies were then treated with 1% BSA in PBST for 30 min to reduce background, followed by anti-PGP9.5 raised in rabbits against human PGP9.5 antigen (1∶100; AbD Serotec, Raleigh, NC, USA) at 4°C overnight, and washed with PBS. Subsequently, the colonies were incubated with anti-rabbit Alexa 568 (1∶500; Invitrogen) for PGP9.5 antibody. Cell nuclei were stained with 4′,6′-diamidino-2-phenylindole staining solution (DAPI; Vector Laboratories, Burlingame, CA, USA) and observed with an excitation filter of 450–560 nm by using a fluorescence microscope (Nikon, Tokyo, Japan) at a magnification of ×200.

### Alkaline phosphatase staining

Alkaline phosphatase (AP) staining was performed using a CBA-300 AP staining kit (Cell Biolabs, San Diego, CA, USA) according to the manufacturer's instructions. Briefly, the cells were fixed in fixation solution, washed three times with PBS containing 0.2% tween-20, and incubated with AP staining solution for 20–30 min. The AP solution was removed, the cells were washed with PBS, and the AP-stained cells were observed by light microscopy (Nikon, Tokyo, Japan) at a magnification of ×200.

### RT-PCR and real-time PCR

Total RNA from collected colonies and 2-month-old beagle testes (2 MOBT) was isolated using the RNeasy Mini Kit (Qiagen, Venlo, the Netherlands). cDNA templates were prepared from 1000 ng total RNA by using the Maxime RT Premix kit (Intronbio, Seongnam, Korea). cDNA synthesis conditions were as follows: 1 cycle each of 60 min at 94°C. cDNA synthesis inactivation was performed under heating condition (5 min at 95°C). Gene-specific primers (Table 1) for glyceraldehyde 3-phosphate dehydrogenase (*GAPDH*), *GFRα-1*, *PLZF*, *Oct4*, *Nanog*, luteinizing hormone receptor (*LHR*), and GATA-binding protein 4 (*GATA4*), were designed from the data retrieved from the NCBI gene database (http://www.ncbi.nlm.nih.gov/); forward and reverse primers were designed in different exons to avoid genomic contamination. RT-PCR was performed with the primers using Mycycler Personal Thermal Cycler (Bio-Rad, Hercules, California, USA); cycling conditions were 30 cycles each of 1 min at 94°C, 1 min at 60–62°C, and 2 min at 72°C for all genes (Table 2). Real-time PCR was performed with the primers using Rotor-Gene Q (Qiagen, Venlo, the Netherlands); cycling conditions of real-time PCR were 40 cycles each of 20 sec at 94°C, 20 sec at 60–62°C, and 20 sec at 72°C for all genes (Table 2). PCR products of all genes were detected by electrophoresis in a 1.5% gel with Tris-acetate-EDTA buffer.

**Table 2:** Primers used for RT-PCR.

Gene symbol, (Accession no.)	Primer sequence	Annealing Temp. (°C)	Methods
*GAPDH*, (NM_001003142)	Forward-CTTCACCACCATGGAGAAGG Reverse- CAGCTCAGGGATGACCTTGC	60	RT-PCR and realtime PCR
*PGP 9.5*, (XM_536245.4)	Forward-ACTCCTGTGGTACCATCGGG Reverse- TTCTCTGCAGACCTTGGCGG	62	RT-PCR and realtime PCR
*GFR-α1*, (XM_005637763.1)	Forward-CACCACCAGCATGTCCAACG Reverse-CCAATAAGCCCTGAGTAGGC	60	RT-PCR and realtime PCR
*PLZF*, (XM_005619755.1)	Forward-ACTGTGCTGGCCTGTACCAG Reverse-TGCAGCCACACTGGCATACC	62	RT-PCR
	Forward-TAACGAGGCTGTGGAGCAGC Reverse- TGTGTCTCCAGGGCATCCTC	62	realtime PCR
*OCT4*, (XM_538830.1)	Forward-GCCGGACAAGGAGAAGCTGG Reverse-CCAGGTTGCCTCTCACTCGG	62	RT-PCR
	Forward-ATATGTGTAAGCTGCGGCCC Reverse-CAATGTGGCTGATCTGCTGC	60	realtime PCR
*Nanog,* (XM_543828.3)	Forward-ACCTCAGTCTCCAGCAGATG Reverse-TCTGACTGTTCCAGGAGTGG	60	RT-PCR
	Forward-TCTGCCACCACGGAATATGC Reverse-TCTGACTGTTCCAGGAGTGG	60	realtime PCR
*GATA4,* (NM_001048112.1)	Forward-GGCACTACCTGTGCAATGCC Reverse-CCTCCTGCCAGCAGTGCTTC	62	RT-PCR and realtime PCR
*LHR*, (NM_001003121.1)	Forward-GGTATGCTGGAGAGTTCCTC Reverse- GATGAAGAGGCAGCTGAAGG	60	RT-PCR
	Forward-CTGGACCACAGTTGTACATC Reverse- ATGAAGGACTCGTGTCAGAG	58	realtime PCR

### Statistical analysis

Diameters of 50 rounded seminiferous tubules were measured in testis sections of 2-, 3-, and 12-month-old beagles by using Stereo Investigator Version 7.5 (MicroBrightField, Williston, VT, USA). PGP9.5-positive spermatogonia were counted on 20 images of testis sections of 2-, 3-, and 12–month-old beagles at a magnification of ×400. Quantitative gene expressions between colonies and testis tissue were calculated with Ct and delta-delta-Ct obtained on comparison with the GAPDH Ct value. All statistical analyses were performed in a one-way nested analysis of variance (ANOVA) with Tukey test using Graphpad Prism 4. The null hypothesis was rejected when the *P*-value was <0.05.

## Results

### Canine testicular development

**Figure 1:**
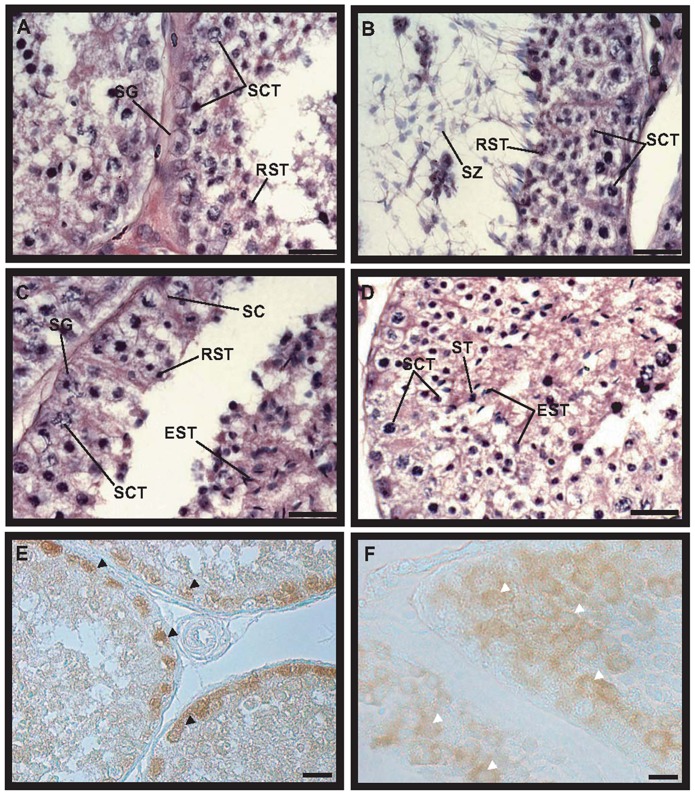
Histological analysis and spermatogonia detection in 12-month-old beagle testes. Panels A, B, C, and D are hematoxylin-eosin–stained testis sections. Spermatogenetic cells are detected in 12-month-old beagle testes. Testis sections were stained with PGP9.5 (E) and DAZL (F) antibodies. Black arrows indicate PGP9.5-positive spermatogonia in panel E and white arrows indicate DAZL-positive spermatocytes in panel F. Scale bars are 20 µm in all panels. EST, elongated spermatid; RST, round spermatid; SC, Sertoli cell; SCT, spermatocyte; SG, spermatogonia; SZ, spermatozoa. Magnification is ×1000 (panels A, B, C, and D) and ×400 (panels E and F).

**Figure 2:**
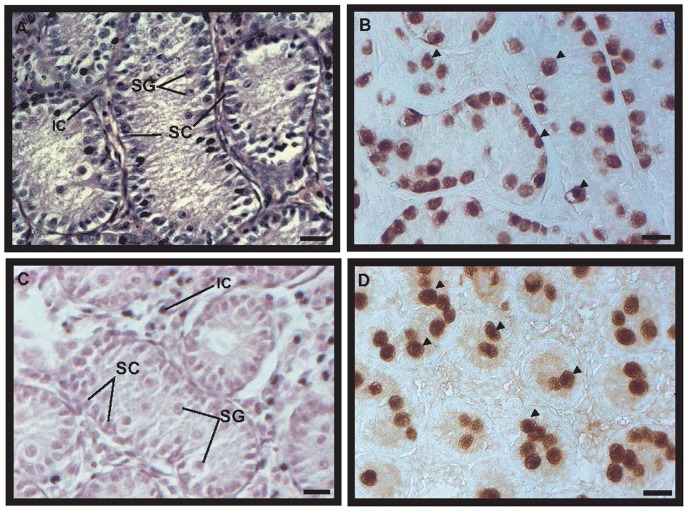
Histological analysis and spermatogonia detection in 3- and 2-month-old beagle testes. Panels A and C are hematoxylin-eosin–stained testis sections in 3- and 2-month-old beagle testes, respectively. Only spermatogonia exist in 3- and 2-month-old beagle testes. PGP9.5-positive spermatogonia are shown in panel B (3-month-old beagle testes) and D (2-month-old beagle testes). Arrows indicate PGP9.5-positive spermatogonia. Scale bars indicate 20 µm in all panels. IC, interstitial cells; SC, Sertoli cell; SG, spermatogonia. Magnification is ×400 in all panels.

**Figure 3:**
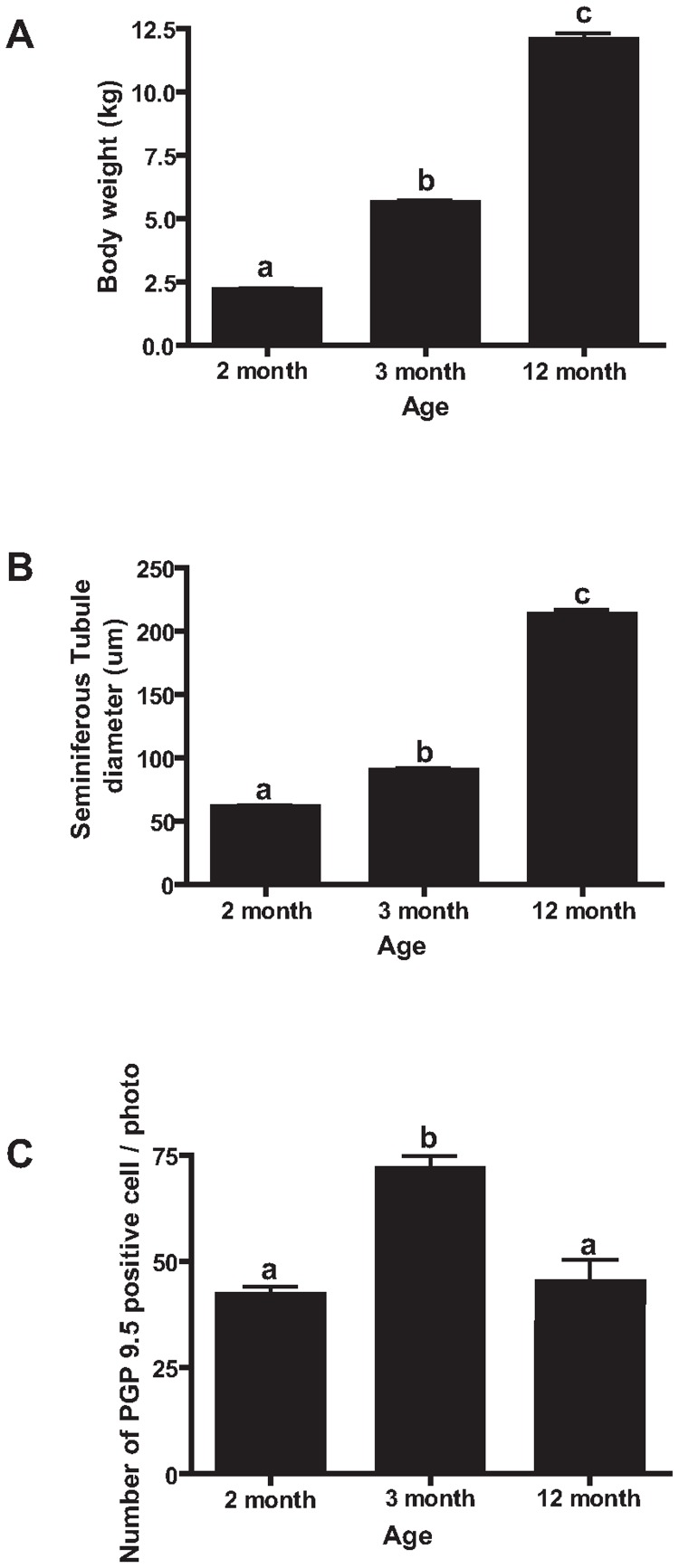
Body weight, size of the seminiferous tubules, and number of PGP9.5-positive spermatogonia in 2-, 3-, and 12-month-old beagle testes. (A) Body weight, (B) seminiferous tubule diameter, and (C) number of PGP9.5-positive spermatogonia. Diameters of rounded seminiferous tubules were calculated by Stereo Investigator Version 7.5, and the numbers of PGP9.5-positive spermatogonia were counted from the images of 2-, 3-, and 12-month-old beagle testis sections taken at ×400 magnification (C). (* P<0.05).

To identify the types of cells in the canine testes during different developmental stages, histological analysis was performed in 2-, 3-, and 12 MOBT. As a result, normal spermatogenesis, containing spermatogonia, spermatocytes, spermatids, and spermatozoa, was observed in 12 MOBT (Fig. 1A–D). However, it was difficult to distinguish the precise developmental stage of spermatocyte, elongated spermatid, and spermatozoa in H&E staining. Based on a previous report [3], we found that stages 1, 5, and 7 of canine seminiferous epithelium cycle were detected (Fig. 1A–D). Spermatogonia were located in the basement membrane of seminiferous tubules (Fig. 1A, C), and spermatocytes were located next to spermatogonia or around the basement membrane (Fig. 1A–D). Round spermatids, elongated spermatids, and spermatozoa were observed close to the lumen of seminiferous tubules (Fig. 1B, D). In immunohistochemistry, we used 9 antibodies that are known spermatogonia markers in mammals. However, only PGP9.5 and DAZL were detected in beagle testes; 7 markers did not react with beagle spermatogonia. PGP9.5 protein was detected only in spermatogonia that exist in the basement membrane of the seminiferous tubules (Fig. 1E). Although DAZL was detected in spermatocytes in the seminiferous tubules, it was not stained around the basement membrane and lumen (Fig. 1F). In 3 MOBT, spermatogonia and Sertoli cells were observed in the seminiferous tubules (Fig. 2A). In 2 MOBT, spermatogonia and Sertoli cells were also observed in seminiferous tubules (Fig. 2C). PGP9.5 was detected in spermatogonia from 3 MOBT, and aligned spermatogonia were located in the basement membrane of the seminiferous tubules (Fig. 2B). In 2 MOBT, PGP9.5 was observed in single and paired spermatogonia in the seminiferous tubules (Fig. 2D). To determine the relationship between growth and spermatogenesis, spermatogonia number, seminiferous tubule diameter, and growth were analyzed. Changes in body weight, seminiferous tubule diameter, and PGP9.5-positive spermatogonia in 2-, 3-, and 12 MOBT are shown in Figure 3. Body weight was significantly increased by the growth of the beagles (Fig. 3A). Interestingly, the body weight of 3-month-old beagles was significantly more than that of 2-month-old beagles (Fig. 3A). The diameter of the seminiferous tubules in 2 MOBT was significantly smaller than that in 3 MOBT, which was smaller than that in 12 MOBT (Fig. 3B). Seminiferous tubule diameter was proportional to the body growth. The number of spermatogonia in a seminiferous tubule of 3 MOBT was significantly higher than in a seminiferous tubule of 2 and 12 MOBT, with the number of spermatogonia in 2 MOBT similar to that in 12 MOBT (Fig. 3C).

### Formation of SDC in different media

**Figure 4:**
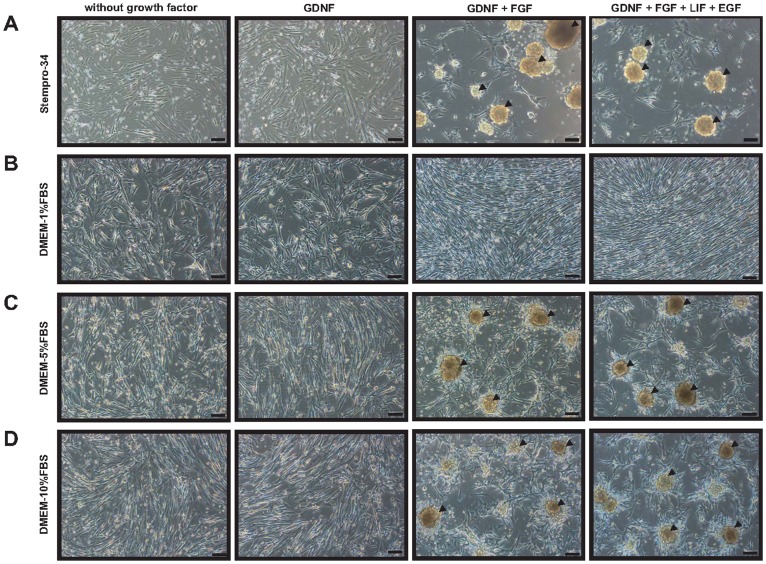
Colony formation in stempro-34 and DMEM-FBS media with growth factors. Total testicular cells were cultured for 7 days in (A) stempro-34, (B) DMEM- 1%FBS, (C) DMEM- 5%FBS, and (D) DMEM- 1%FBS. Combinations of growth factors were added in each media. Arrowheads indicate the colonies formed in specific media conditions. Scale bars indicate 100 µm in all panels. GDNF, glial cell-derived neurotrophic factor; bFGF, basic fibroblast growth factor; LIF, leukemia inhibitory factor; EGF, epidermal growth factor. Magnification is ×100 in all panels.

Two MOBT were selected for the culture of spermatogonia owing to their single and pairwise distribution in the seminiferous tubules, thus providing spermatogonia close to type A spermatogonia. Two different media (stempro-34 and DMEM-FBS) were used for total testicular cell culture to obtain SDCs. When total testicular cells were cultured with stempro-34 medium, colonies were not formed under GDNF-positive and other growth factor-negative conditions at day 7 (Fig. 4A). When bFGF was added in stempro-34 media with GDNF from the first day of culture, colony formation was observed at day 7. The colonies were also formed in stempro-34 media with GDNF plus FGF, and GDNF plus FGF, LIF, and EGF at day 7 (Fig. 4A). No colonies were observed in DMEM with 1% FBS containing growth factors at day 7 (Fig. 4B). Colonies were also not formed in DMEM media with 5% and 10% FBS with GDNF, or without growth factors, at day 7 (Fig 4C, D). Colony formation was observed at day 7 when GDNF plus FGF, and GDNF plus FGF, LIF, and EGF were added to DMEM with 5% and 10% FBS (Fig. 4C, D).

### Characterization of SDCs at day 7 of culture

**Figure 5:**
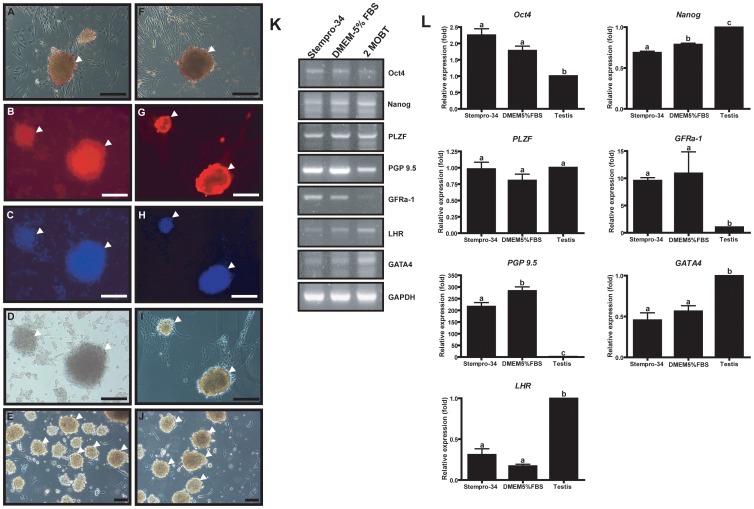
Characterization of colonies cultured in stempro-34 and DMEM-5%FBS media conditions at day 7. Colonies cultured in stempro-34 supplemented with GDNF, FGF, LIF, and EGF (A, B, C, D, and E) and DMEM-5% FBS supplemented with GDNF, FGF, LIF, and EGF (F, G, H, I, and J) were analyzed. AP-staining results are in A and F. Colonies stained with PGP9.5 antibody are in B and G. Nuclear staining is shown in panels C and H. Bright-field images are shown in panels D and I. Collected colonies for PCR analysis are shown in panels E and J. Arrowheads indicate the colonies. RT-PCR and real-time PCR were performed on colonies and 2-month-old beagle testes (panels K and L), respectively, with *OCT4, NANOG, PLZF, PGP9.5, GFRα-1, LHR*, and *GATA4* primers, as shown in Table 2. Magnification is ×200 in panels A–D and F–I, and ×100 in panels E and J. MOBT, month-old beagle testes. (* P<0.05).

AP staining, immunocytochemistry, and RT-PCR and real-time PCR were performed with cells from colonies formed in stempro-34 and DMEM-5%FBS media at day 7. The colonies from both media were positive for AP staining (Fig. 5A, F), and PGP9.5 (Fig. 5B, G). Cell nuclei and normal morphology of the colonies were observed after immunocytochemistry (Fig. 5C, D, H, I). The colonies, which were attached to the bottom of the well, were separated from the bottom using a 1-ml pipette, and floating colonies were collected by pipette to perform RT-PCR and real-time PCR (Fig. 5E, J). Early spermatogonia and stem cell markers, *Oct4, Nanog, PLZF, PGP9.5* and *GFRα-1*, were expressed in colonies from stempro-34 and DMEM-FBS media, and 2 MOBT (Fig. 5K). *GATA4* and *LHR*, which are known as Sertoli and Leydig cell markers respectively, were not detected in colonies from either media (Fig. 5K). Higher *Oct4* expression was detected in colonies cultured in stempro-34 medium and DMEM-FBS than that in 2 MOBT, and *Nanog* expression from colonies in both media was lower than that in 2 MOBT (Fig. 5L). No significant difference in *PLZF* expression was detected in colonies from either media or 2 MOBT (Fig. 5L). Interestingly, *PGP9.5* and *GFRα-1* expression in colonies from both media were significantly higher than that in 2 MOBT (Fig. 5L). In colonies from both media, expression levels of *GATA4* and *LHR* were lower than that in 2 MOBT (Fig. 5L).

## Discussion

The present study is the first to report the investigation of early stage development of canine testes and the in vitro culture system of spermatogonia from canine testes. It has been reported that canine primordial germ cells were observed at days 21–25 of gestation, and are positively stained with anti-OCT4 antibody [6]. In canine spermatogenesis, the seminiferous epithelium cycle has 8 stages, whereas the cycle of seminiferous tubules is 14 stages in rats and 12 stages in mice [1], [7]. Soares and colleagues have reported that 2- to 7-year-old beagles have an 8-stage seminiferous epithelium cycle, and that stages 4 and 5 are the most frequently observed [3]. In our results, stages 1 and 5, which contain spermatogonia, spermatocyte, spermatid, and spermatozoa, were observed in 12 MOBT, indicative of mature testes. In neonatal mice, seminiferous tubule area increases with body growth, with 6- to 10-day postnatal testes containing only spermatogonia, and the first meiotic division into spermatocytes occurring in 14- to 20-day postnatal testes [18]. Our results showed that the diameter of the seminiferous tubules increased proportionally to the body weight. However, the first meiotic division did not occur until 2–3 months postpartum. These results indicate that an increase in spermatogonia number does not occur with the growth of the seminiferous tubules until 3 months of age. It is noteworthy, that single and paired spermatogonia were abundant in 2 MOBT, whereas aligned spermatogonia were only observed in 3 MOBT, indicating that 2 MOBT is the optimal source for collecting early type spermatogonia. In this study, we found that specific body weight is correlated with early stages of spermatogonia development. A previous study revealed that body length affects gonadal development in both sexes in pufferfish [19]. This data is also consistent with our previous reports in porcine, showing that abundant spermatogonia are found in 5-day-old pig testes [20], and isolated spermatogonia from this age testes are successfully maintained in an in vitro culture system.

In vitro culture of SSCs is important for exploration of SSCs self-renewal, differentiation, and manipulation. SSC cultures longer than 2 months have not been established for domestic animals, although short term SSC cultures have been reported in goat, pig, and cattle [9]. Thus far, two kinds of media (stempro-34 and DMEM-FBS) have been used for SSCs culture in domestic animals. Colony formation has been observed in SSCs culture of goat and pig by using DMEM-FBS medium, and these colonies contain PGP9.5-positive cells [21], [22]. Colonies were formed in SSC cultures of piglets and calves by using stempro-34 medium (serum-free medium) and contained Dolichos biflorus agglutinin (DBA)-positive cells [23], [24]. In our previous report, SDC have been observed in the culture of porcine testicular cells that contain PGP9.5-positive cells with stem and germ cell characteristics [17]. In addition, growth factors are essential to form the SDCs in SSC cultures. In rodents, colony formation, which were cultured on SIM mouse embryo-derived thioguanine and ouabain resistant (STO) feeders in a serum-free defined medium by using THY1-positive SSCs, requires GDNF and bFGF for SSC cultures, and proliferation in hamster germinal stem cells was induced by GDNF plus FGF2, but not by GDNF plus EGF [25], [26]. In pig SSC cultures, EGF and FGF had a positive effect on the number and size of the SSC-like colonies, and addition of EGF and FGF to the primary cell cultures of neonatal pig testes affected the expression of NANOG, PLZF, OCT4, and GATA4 [23]. In addition, FGF2 also mediated mouse SSC self-renewal via up-regulation of Etv5 and Bcl6b through MAP2K1 activation [27]. These reports imply that SSC colonies can be formed in stempro-34 and DMEM-FBS media and that FGF has an important role in SSC cultures. In the present study, the colonies were observed in both media at day 7, and the addition of FGF considerably affected the colony formation from 2 MOBT. These results indicate that stempro-34 and DMEM-FBS media with the addition of GDNF and FGF are well suited for preparing SDC derivation from neonatal beagle testes.

When the culture period was extended to 21 days, the colonies detached from the feeder cells at days 14 and 20, and the size of colonies increased more when the colonies clustered together at day 14 in stempro-34 medium (Fig. S1A, B). In DMEM with 5% FBS containing growth factors, feeder cells were more confluent by day 7, and the colonies were tightly attached to feeder cells from days 14 to 20. The size of colonies increased from days 14 to 20; however, newly developed colonies were not identified (Fig. S1D, E). Alkaline phosphatase (AP) staining results showed that the colonies cultured in stempro-34 media are AP-positive, although colonies in DMEM media did not react to AP staining (Fig. S1C and F, respectively). In our previous study of porcine spermatogonia, similar results were observed regarding the detachment of pSGC colonies from feeder cells in long-term culture at 31°C in stempro-34 media [17]. This might be related to the aging of feeder cells, and the aged feeder cells could not provide a sufficient survival environment for SDCs. However, in DMEM with FBS media, increase in the number of feeder cells from 14 to 20 days of culture may be attributed to the FBS effect. After formation of SDCs, aged feeder cells that may no longer support germ cells were denaturalized and proliferated by FBS; therefore, they did not provide a sustainable environment for SDCs, and SDCs lost their own characteristics, such as AP reactivity.

Recently, many spermatogonia markers have been studied in rodents [5]. NANOG, OCT4, and SOX2 cooperatively maintain the regulatory network responsible for self-renewal and pluripotency, and are often used as stem cell markers [28]. There are few reports for OCT4 and NANOG mRNA expression in SSCs and SDCs; higher expression of both OCT4 and NANOG mRNA was detected in porcine SDCs [17]. However, the expression of both proteins in spermatogonial germ cells remains unclear. OCT4 was expressed in cultured porcine germ cell colonies, but NANOG was not expressed [23]; furthermore, OCT4 was expressed in cultured mouse germinal stem cells under serum- and feeder-free conditions, but NANOG was not expressed [29]. NANOG, but not OCT4, has been detected in porcine neonatal testes [30]. Our present results correlate with reports on cultured mouse germinal stem cells; therefore, it is possible that *Oct4* and *Nanog* expression levels vary in SDCs and SSCs of each species. In addition, mRNA expression of *PGP9.5* and *GFRα-1*, as canine germ cell markers, were expressed at significantly higher levels in cultured colonies than in total cells of neonatal beagle testes. These results suggest that the colonies cultured in stempro-34 and DMEM-FBS media are spermatogonial germ cell clusters with stem cell characteristics.

In conclusion, spermatogonia of 2 MOBT, located in the seminiferous tubules as single and paired cells, can be used for the derivation of SDC. FGF plays a key role in SDC formation from beagle testes, and these colonies express genes that are characteristic of germ and stem cells.

## Supporting Information

Figure S1 **Extended culture of SDCs in stempro-34 and DMEM-5%FBS with GDNF, FGF, LIF and EGF.** Culture of SDCs was extended until day 21 in stempro-34 (A and B) and DMEM- 5%FBS (D and E) condition. Arrowheads indicate the colonies (A–F). Black arrowheads indicate floated colonies at day 14 in panel A, and clustered colonies at day 21 in panel B and C. White arrowheads indicate tightly attached colonies at day 14 and 21 in panel D, E and F. AP staining results of colonies from stempro-34 and DMEM-5%FBS are presented in panels C and F, respectively. Scale bars indicate 100 µm in all panels.
(TIF)
