# Supplementary figures and images for: Identification and In Vitro Derivation of Spermatogonia in Beagle Testis
Source: PLoS One. 2014 Oct 15;9(10):e109963. doi: 10.1371/journal.pone.0109963 (PMC4198177; doi:10.1371/journal.pone.0109963)

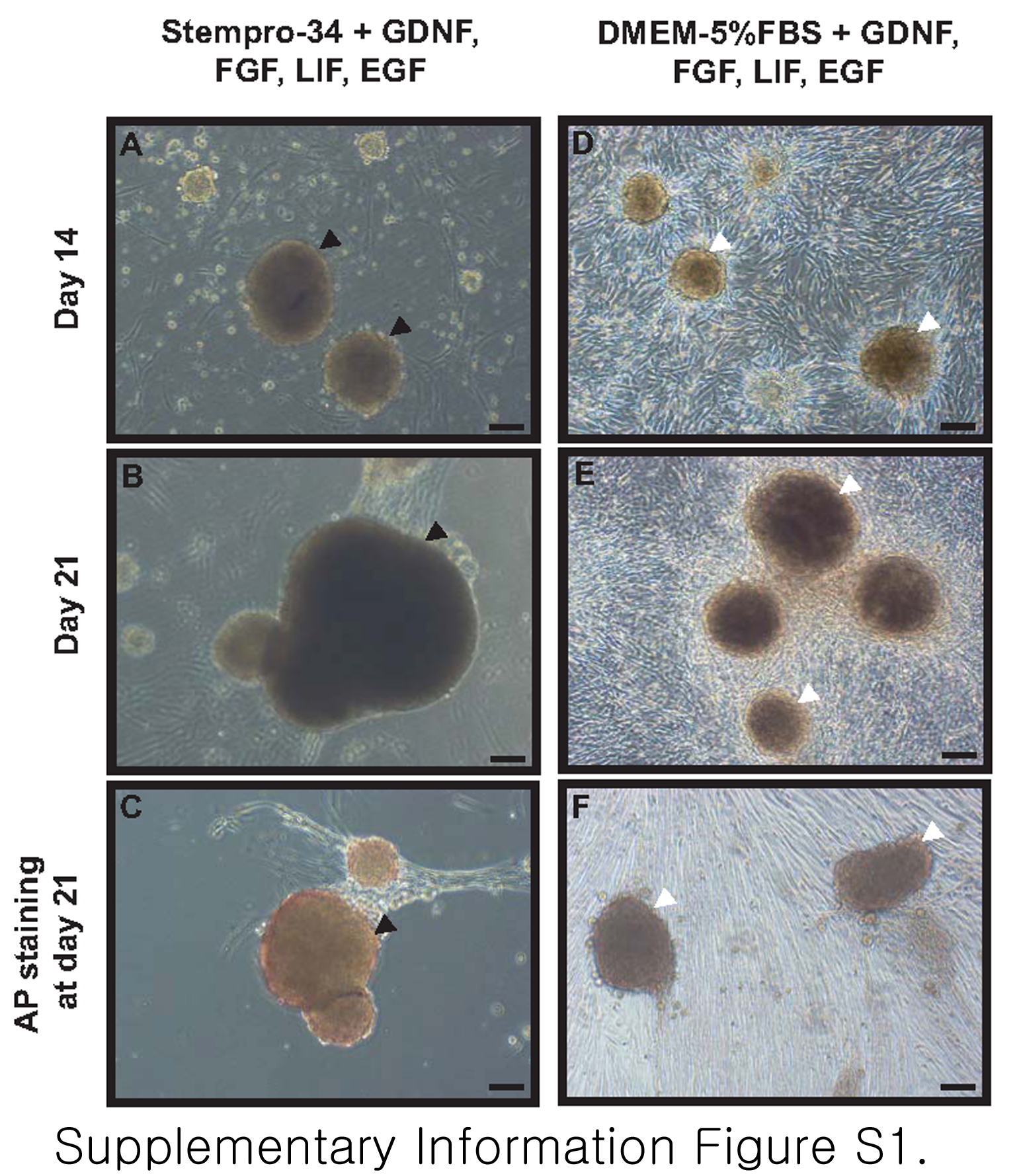

Supplement: Figure S1 — Extended culture of SDCs in stempro-34 and DMEM-5%FBS with GDNF, FGF, LIF and EGF. Culture of SDCs was extended until day 21 in stempro-34 (A and B) and DMEM- 5%FBS (D and E) condition. Arrowheads indicate the colonies (A–F). Black arrowheads indicate floated colonies at day 14 in panel A, and clustered colonies at day 21 in panel B and C. White arrowheads indicate tightly attached colonies at day 14 and 21 in panel D, E and F. AP staining results of colonies from stempro-34 and DMEM-5%FBS are presented in panels C and F, respectively. Scale bars indicate 100 µm in all panels. (TIF) [file pone.0109963.s001.tif]
